# A super-giant basal cell carcinoma of the scalp

**DOI:** 10.1093/omcr/omae149

**Published:** 2024-12-10

**Authors:** Alexandros Patsouras, Nikolaos Garmpis, Eleni I Effraimidou, Dimitrios Dimitroulis, Dimitrios Papoutsas, Iason Psilopatis, Anna Garmpi, Evangelos Diamantis, Kleio Vrettou, Sampaziotis Dimitrios, Paraskevi Ioanna Tasioula, Christos Damaskos

**Affiliations:** Second Department of Pulmonology, Sotiria General Hospital, 152 Messogeion Ave 11527, Athens, Greece; Department of Surgery, Sotiria General Hospital, 152 Messogeion Ave 11527, Athens, Greece; N.S. Christeas Laboratory of Experimental Surgery and Surgical Research, Medical School, National and Kapodistrian University of Athens, Agiou Thoma 17 11527, Athens, Greece; First Surgical Department, University Hospital of Alexandroupolis, Democritus University of Thrace, Dragana, Dragana 68100, Alexandroupolis, Greece; Second Department of Propedeutic Surgery, Laiko General Hospital, Medical School, National and Kapodistrian University of Athens, Agiou Thoma 17 11527, Athens, Greece; Department of Surgery, Sotiria General Hospital, 152 Messogeion Ave 11527, Athens, Greece; Department of Obstetrics and Gynecology, University Erlangen Hospital, Universitätsstraβe 21-23, 91054, Erlangen, Germany; First Department of Propedeutic Internal Medicine, Laiko General Hospital, Medical School, National and Kapodistrian University of Athens, Agiou Thoma 17 11527, Athens, Greece; Academic Department of Internal Medicine - Endocrinology Unit, Agioi Anargyroi General Oncology Hospital of Kifisia, National and Kapodistrian University of Athens, Kalyftaki 14564, Athens, Greece; Department of Cytopathology, Sismanogleio General Hospital, Sismanogleiou 1 15126, Marousi, Athens, Greece; Department of Pathology, Sismanogleio General Hospital, Sismanogleiou 1 15126, Marousi, Athens, Greece; Department of Surgery, Sotiria General Hospital, 152 Messogeion Ave 11527, Athens, Greece; N.S. Christeas Laboratory of Experimental Surgery and Surgical Research, Medical School, National and Kapodistrian University of Athens, Agiou Thoma 17 11527, Athens, Greece; Deparment of Emergency Surgery, Laiko General Hospital, Agiou Thoma 17 11527, Athens, Greece

**Keywords:** basal cell carcinoma, super-giant, skin, cancer

## Abstract

Basal cell carcinoma is a malignant skin cancer, originating from basal cells. However, it is regarded more benign than other skin cancers, in the majority of the cases. If left untreated, it can lead to various complications, degradation of quality of life and even mortality to the patient. A basal cell carcinoma with one dimension more than 20 cm, is defined as super-giant. In this report, we present a case of a super-giant basal cell carcinoma occupying most of the scalp in an elderly patient, causing him severe anemia and general malaise.

## Introduction

Basal cell carcinoma (BCC) is regarded a skin cancer which originates from basal cells [1]. Its location varies, with most of the cases to be found in the face, trunk and extremities [2]. It is not regarded as an aggressive cancer since it has slow metastatic and growth abilities. However, if left untreated, it can grow in size and cause local and systemic complications to the patient. BCCs greater than 5 and 20 cm in size are regarded as giant and super-giant, respectively [3–6]. These BCCs account for less than 0.5% of BCC [7]. Histopathologically, according to the world health organization (WHO) classification, BCCs are divided into ten subtypes (Table 1) [8].

**Table 1:** Histopathological classification of basal cell carcinomas according to world health organization (WHO)

**Histopathological subtype**		**Recurrence risk**
1	Nodular	Low
2	Superficial	
3	Pigmented	
4	Infundibulocystic	
5	Fibroepithelial	
6	Basosquamous carcinoma	High
7	Sclerosing/Morpheaform	
8	Infiltrating	
9	BCC with sarcomatoid differentiation	
10	Micronodular	

BCC: Basal cell carcinoma.

Herein, we report a case of a super-giant BCC in the head of an elderly man, which led to severe complications.

## Case presentation

**Figure 1:**
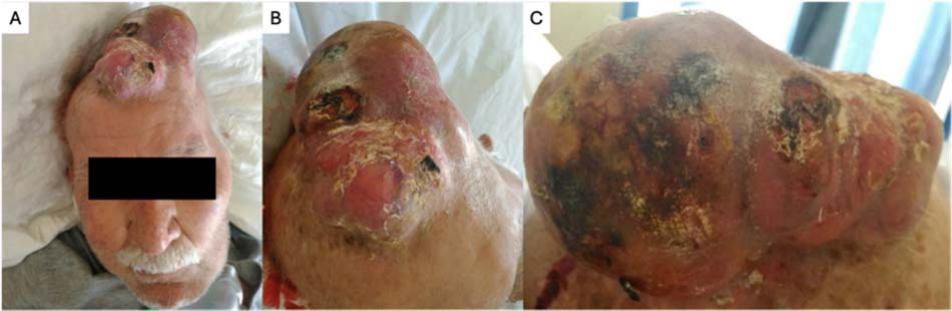
(**A** and **B**) Macroscopic view of a super-giant basal cell carcinoma of the scalp.

**Figure 2:**
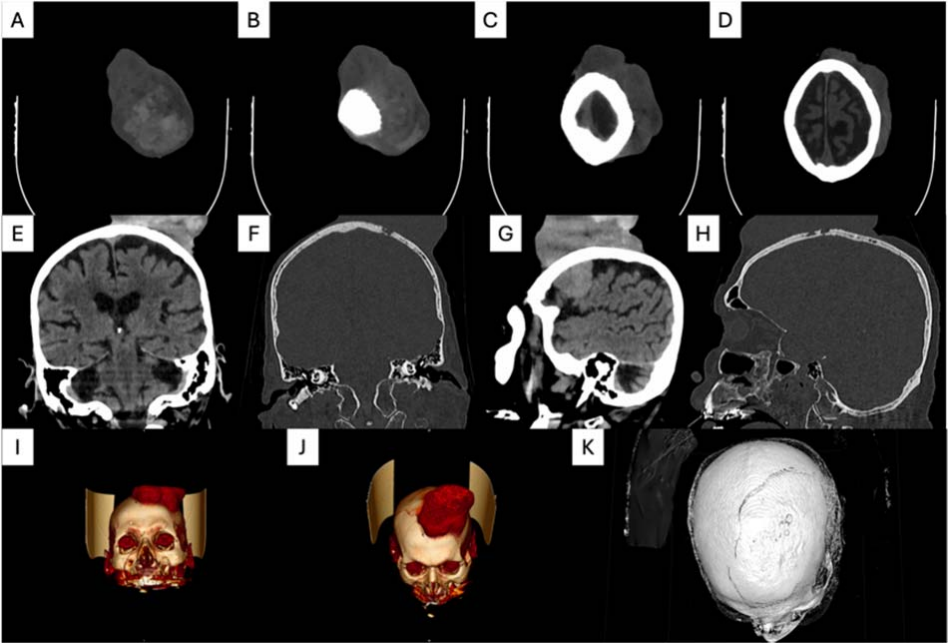
Computed tomography findings: An enhanced heterogeneous mass extending above the scalp exerting compressive effects on the brain and concomitant diffuse bone infiltration. (**A**–**D**) Axial view; (**E** and **F**) Coronal view; (**G** and **H**) Sagittal view; (**I**–**K**) 3-D reconstruction.

**Figure 3:**
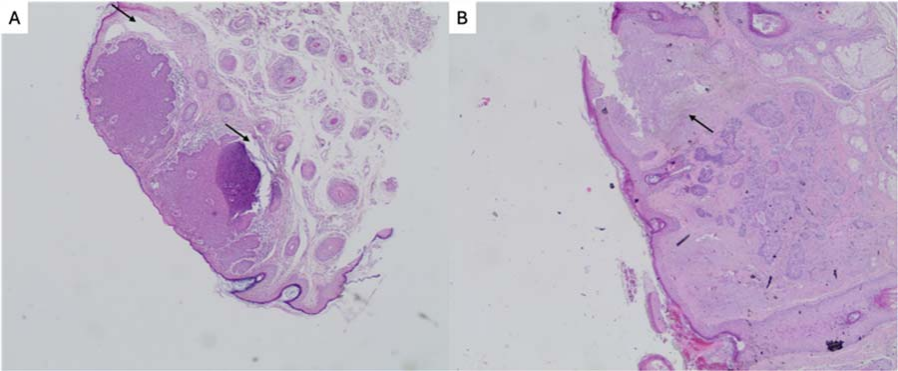
Histopathological findings of mixed basal cell carcinoma including nodular and micronodular subtypes. (**A**) Two relatively circumscribed masses with epidermal attachment, consisting of large basaloid lobules with peripheral nuclear palisade. Cleft formation (or retraction artifact) between tumor lobules and stroma is also apparent (arrow). H&E ×400; (**B**) Small basaloid nests diffusely infiltrating the dermis with peripheral palisading less prominent than in nodular BCC. The retraction artifact is absent. There is also solar elastosis next to the lesion (arrow). H&E ×400.

An 88-year-old male presented to our department due to a huge skin lesion, occupying a big part of his scalp. He complained about weakness, shortness of breath and general malaise. His medical history included hypertension and diabetes mellitus type 2. He was an active smoker with a history of a 50 pack/years. He was a farmer. Upon presentation, the vital signs were abnormal. His blood pressure was 145/97 mmHg, 78 beats/min, tachypneic and afebrile. His oxygen saturation was 92% on room air. He had a necrotic, hemorrhagic, giant lesion. In addition, bone infiltration was observed. The dimensions were 20.5 × 11.6 × 7 cm. This mass was lobulated in shape, with ulcers, red-yellow-purplish in color. Fluid and blood were leaking. The mucosa was very fragile and bleeding upon palpation. Vessels were visible on the surface (Fig. 1A–C). The disease was extremely progressed as the patient delayed seeking medical help. This lesion had grown progressively over the previous three years. This lesion was unique, and the rest of the skin examination was normal. His laboratory exams revealed an anemia (Hb value at 7,4 g/dl, ferritin levels at 12 ng/ml, and an iron binding capacity of 355 mcg/dl). A computed tomography (CT) of the neck, chest and abdomen showed no metastatic lesions. A CT of the head showed diffuse bone infiltration and an enhanced heterogeneous mass extending above the scalp (Fig. 2A–K). The mass was exerting compressive effects on the brain. Biopsies were excised from the affected skin. The histopathological examination demonstrated pleiomorphic, columnar and cuboid cells with keratin spots and fibrous stroma. The diagnosis was a mixed BCC including nodular and micronodular subtype (Fig. 3A and B). Neither radiochemotherapy nor surgery were offered to the patient. Only palliative treatment was administrated due to his poor clinical condition and his will, including transfusion and local hemostasis of the lesion. The patient suffered from nosocomial pneumonia and died in the hospital a few days ago.

## Discussion

A few cases of super-giant BCC have been reported in the international literature so far [4–7, 9–24]. These lesions can be encountered in various locations, most of them in the trunk [6]. In very few cases, including ours, this lesion extends in the majority of the scalp (Table 2) [14–16]. Reasons for the development of these BCC are exposure to sun light, previous BCC in the same spot, neglection and immunosuppression [7, 25]. Our patient was an elderly farmer with diabetes mellitus type II. Thus, exposure to sunlight and immunosuppression existed. Super-giants BCCs are by far more aggressive, with greater local infiltration, metastatic ability, and recurrence [3, 6, 18]. Lungs constitute the primary sites of metastasis. In tumors greater than 25 cm, metastasis is regarded as very possible [7, 10, 15, 17–19, 21–23]. In our case, bone infiltration and extrinsic pressure of the brain occurred. However, no distant metastases were found. In addition, severe chronic or acute anemia, as in our case, has been reported in three similar cases of giant BCC [4, 14, 18]. Histopathologically, two cases of head super-giant BCC had nodular subtype and one morpheaform. Our patient is the unique with the rare coexistence of nodular and micronodular subtype.

**Table 2:** Reported cases of super-giant basal cell carcinoma in the scalp.

**Study**		**Age/Sex**	**Size**	**History**	**Histopathology**	**Metastasis**	**Treatment**	**Follow up**
1	Andersen *et al*. [16]	73/F	25 × 20 cm	15 y	Nodular	Exposure of soft tissue and bone	Radiation, Vismodegib	Total: 40 m. Bleeding and vision loss 6 m after radiation. Satisfying results after 20 m of vismodegib therapy.
2	Yoham *et al*. [15]	52/M	21 × 16 cm	11 y	Morpheaform	Exposure of soft tissue and bone	No	Vision loss 1 m after diagnosis.
3	Okano *et al*. [14]	71/M	NR	10 y	Nodular	No	Surgery	Recurrence after 5 m and re-operation. 6 m later free of disease.
4	Current study	88/M	20,5 × 11,6 cm	3 y	Mixed: Nodular and micronodular	Exposure of soft tissue and bone	No	Death in few days from other reason.

F: Female; y: Years; m: Months; M: Male; NR: Not reported.

The prompt management of this clinical entity is wide surgical treatment with clear margins between 5 mm and 1 cm [24]. If possible, plastic surgery should then be performed [14, 20, 24, 26, 27]. Another option for locally advanced, inoperable, or even metastatic BCC is chemotherapy [28]. Unfortunately, in our case, surgery or (radio-)chemotherapy were not an option due to the bad clinical condition. Radiotherapy was not scheduled due to patient’s will.

In conclusion, this case highlights possible complications which can occur from neglected BCC. BCC is a non-aggressive type of skin cancer, which can easily be treated. However, if left untreated, general malaise, severe anemia due to blood loss, and dyspnea due to severe anemia can manifest.

This case is unique due to the extremely rare presentation of a super-giant BCC on the scalp, characterized by its massive size, the coexistence of nodular and micronodular subtypes, and significant complications like bone infiltration and severe anemia. The patient’s history of chronic sun exposure, diabetes, and delayed medical intervention contributed to the tumor’s aggressive behavior. The case emphasizes the critical importance of early detection and treatment of BCC, even though it is typically non-aggressive, to prevent severe local and systemic complications, highlighting the need for greater awareness and timely intervention in similar cases.
